# Trapeziometacarpal Dislocations in Pediatric Age, Is There a Better Treatment? Series of Cases and a Systematic Review

**DOI:** 10.3390/jcm13082197

**Published:** 2024-04-11

**Authors:** Pablo Martin-Diaz, Laura M. Perez-Lopez, Diego Gutierrez-de la Iglesia, Beatriz Miron-Dominguez, Enric Domínguez, Miguel Perez-Abad

**Affiliations:** 1 Hospital Sant Joan de Déu Barcelona, Sant Joan de Déu 2, 08950 Esplugues de Llobregat, Spain; pablomartin.diaz@sjd.es; 2 Institut de Recerca Sant Joan de Déu, Santa Rosa 39-57, 08950 Esplugues de Llobregat, Spain; 3 Hospital Trueta, Avinguda de França S/N, 17007 Girona, Spain; 4 Institut de la Ma, Carrer Pedro i Pons 1, 08195 Sant Cugat del Valles, Spain; beatriz.miron.dominguez@gmail.com; 5 PSMAR Hospital del Mar, Ciutat Vella, 08003 Barcelona, Spain; edominguezfont@psmar.cat; 6 Kaplan Hand Institute, Av. de Josep Vicenç Foix, 71, 08034 Barcelona, Spain; 7 Upper Limb Surgery Unit, Orthopaedic and Traumatology Department, Consorci Sanitari del Maresme, Hospital de Mataró, Carretera de la Cirera 230, 08304 Mataro, Spain

**Keywords:** carpometacarpal joints, joint dislocations, child, adolescent, surgery, closed fracture reduction, cast, surgical, recurrence, thumb, trapezoid bone

## Abstract

(1) **Background**: Dislocations of the trapeziometacarpal joint (TMC) are uncommon in children and adolescents. Only a few isolated cases are reported in the literature. Therapeutic guidance is minimal and inconclusive. (2) **Methods**: The authors present four patients treated for this unusual lesion. We evaluated the evolution according to treatment, age, patient activity, and quickDASH. Despite the clear limitation of the small number of patients, it is relevant to try to better understand this lesion and its evolution. A systematic review of the literature was also conducted. (3) **Results**: This is the largest published series of TMC dislocations in children and adolescents. Patients included a 12-year-old girl treated conservatively with a poor quickDASH; a 9-year-old girl treated surgically with the Eaton–Littler technique for a new dislocation with a partially modified quickDASH; a 13-year-old boy with two necessary closed reductions for a new dislocation and a very good final quickDASH; and a 12-year-old boy treated with closed reduction and percutaneous fixation with excellent final results with quickDASH. (4) **Conclusions**: In the absence of scientific evidence, conservative treatment and ligament reconstruction did not provide good functionality. In contrast, closed reduction with percutaneous fixation provided excellent results. Therefore, the authors would recommend closed reduction and percutaneous needle fixation as an elective method to treat TMC dislocations in pediatric and adolescent patients.

## 1. Introduction

Trapeziometacarpal (TMC) dislocations account for less than 1% of hand injuries in adults [1] and are even rarer in children and adolescents [2]. In adults, the causes are trauma, rheumatoid arthritis, or nerve injury associated with paralysis [3]. In children, however, trauma and/or laxity are the two most common causes.

**Figure 1:**
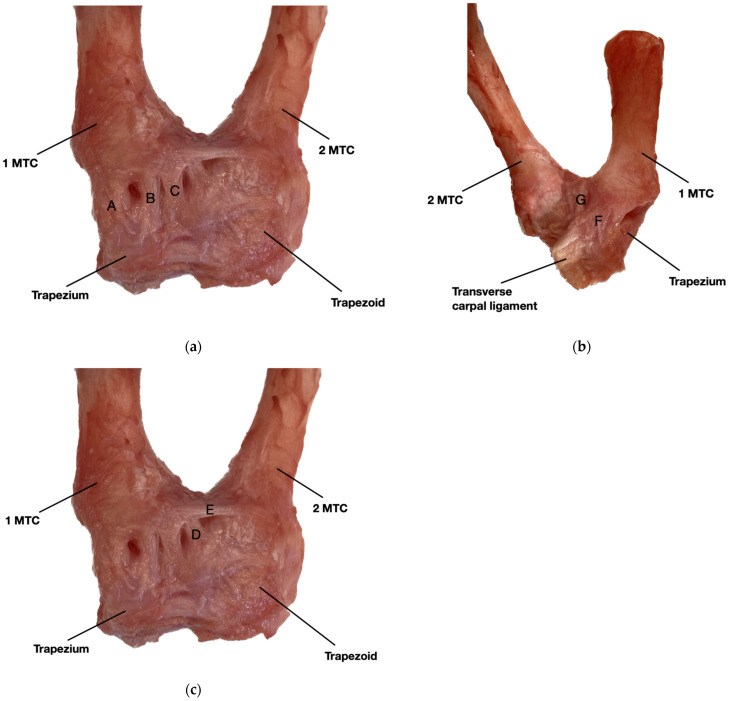
Anatomical dissection of the ligamentous structures of the TMC joint. Author: Enric Dominguez-Font. (**a**) Dorsal view of the dorsal ligament group of the TMC joint: (A) dorsoradial ligament (DRL), (B) dorsocentral ligament (DCL), and (C) posterior oblique ligament (POL). (**b**) Volar view of the volar ligament group of the TMC joint: (F) anterior oblique ligament (AOL) and (G) ulnar colateral ligament (UCL). (**c**) Dorsal view of the ulnar ligament group of the TMC joint: (D) dorsal trapeciometacarpal ligament (dTMCL) and (E) intermetacarpal ligament (IML).

In order to manage these injuries satisfactorily, it is essential to know the anatomy of the TMC joint (Figure 1a–c) and to understand its pathophysiology, aspects that have been extensively described in the literature.

At least 16 ligaments are known to surround the TMC joint [4].

We know that static stability is primarily provided by the joint capsule and five ligaments [5]: the dorsoradialis ligament (DRL), the anterior oblique ligament (AOL) or palmar peak ligament, the posterior oblique ligament (PDA), the anterior trapeziometacarpal ligament or ulnar collateral ligament (UCL), and the intermetacarpal ligament (IML) (Figure 1a–c). However, the importance of each of these ligaments is controversial. Some authors consider the anterior oblique ligament (AOL) to be the main stabilizer of the MCL [6,7]. In the same vein, Pellegrini [8] observed that degeneration of the AOL allows increased forces through the joint, leading to progressive joint laxity and subluxation of the metacarpal over the trapezium. In contrast, other studies suggest that the dorsal complex (DRL, DCL, and POL) is the fundamental stabilizing element [9]. Lamas [10] attributed a greater role to the DRL portion. Strauch [11] also mentions the importance of the DRL as the primary stabilizer in preventing acute MCL dislocation. A recent study has suggested that the dorsal ligaments, considered to be static stabilizers, are actually a structure composed of the aponeurosis of the first dorsal interosseous muscle and the joint capsule [12]. The IML has also been suggested as a fundamental stabilizer of dorsoradial instability [13]. Activation of the opponens pollicis has also been shown to play a key role in stabilizing the TMC [12].

Ladd [14] provides relevant information on the relationship between ligamentous anatomo-histological features and the role of these ligaments in the joint stability of the TMC. According to their study, the dorsal deltoid ligament complex is uniquely strong. It is the thickest and histologically most cellular, and has the highest number of sensory nerve endings. In contrast, the anterior oblique ligament is thin and hypocellular, structurally more like a capsular structure, and inconsistent in location.

According to Edmunds [5], the two main stabilizers of the TMC joint during impingement and pronation are the volar border of the first metacarpal of the thumb and the dorsal radial ligament complex. Thus, in the final phase of thumb opposition, known as screw torque, the rotation of the metacarpal projection of the thumb in the pivot area of the trapezius notch and the tension of the dorsal ligament provide stability to the TMC joint during pinch and grasp movements. The IML also plays an important role in neutralizing a second force on the first interdigital space of the thumb, preventing separation between the first and second metacarpal bases. According to their findings, the anterior trapeziometacarpal ligament or UCL plays a very limited role in the stability of the TMC.

In most cases, the mechanism of injury appears to involve forced flexion of the first metacarpal [15].

Treatment in adults includes closed reduction and needle fixation with open reduction and ligamentous reconstruction, usually using the flexor carpi radialis tendon [7,16,17]. In the pediatric age group, the open technique carries a risk of physical injury to the base of the first metacarpal [3]. At present, the literature seems to recommend it only in the exceptional case of recurrent dislocation with chronic instability following acute injury [2]. On the other hand, there is a paucity of literature and, therefore, no consensus on the best therapeutic technique for the management of MCL dislocation in children and adolescents.

## 2. Materials and Methods

This project involved two methodologies. Firstly, for the proper conduct of the systematic review, we followed the PRISMA 2020 Statement (checklist and flow chart) [18]. Authors have not registered.

The literature review was conducted using three databases, PubMed, Cochrane, and ClinicalKey, from the start of the study to January 2024. Inclusion criteria were traumatic dislocations of the TMC joint in patients up to 17 years of age. Exclusion criteria were physeal dislocation fractures with undislocated first metacarpal base epiphysis, and age 18 years or older. Three reviewers, pediatric upper limb surgeons, worked independently.

For each article reviewed as meeting the inclusion criteria, data were found on the number of patients presented, the time from injury to treatment, the therapeutic method applied, the results in terms of subsequent stability, and subjective patient sensation, as well as the follow-up time. On the other hand, no information was found on the final functionality quantified with scales such as quickDASH, or, sometimes, the treatment defined in detail. In this sense, it is important to highlight the importance of quantifying the functional results in order to gain a deeper understanding of the evolution of this pathology, as well as to try to correlate these results with previous variables such as the type of treatment.

Secondly, patients treated for trapeziometacarpal dislocation in the pediatric upper extremity surgery unit of the pediatric orthopedic surgery and traumatology department of a national referral children’s hospital between 2013 and 2024 were prospectively recruited. Their medical records were reviewed. All patients signed informed consent forms for the present study. In children under 14 years of age at the time of recruitment, the informed consent form was also signed by the parent or legal guardian.

Development was assessed clinically in terms of thumb opposition using the Kapandji test [19] with interphalangeal joint extension [20], metacarpophalangeal and interphalangeal flexion, fine and lateral pincers, and TMC stability.

TMC joint instability was assessed from the patient’s history. Pain or weakness at the base of the thumb, particularly with fine grip and holding heavier objects, was reported. We also clinically assessed TMC stability in neutral, radial abduction, palmar abduction, and thumb opposition.

For proper radiographic evaluation of the TMC joint, the following plain radiographs were obtained

**Figure 2:**
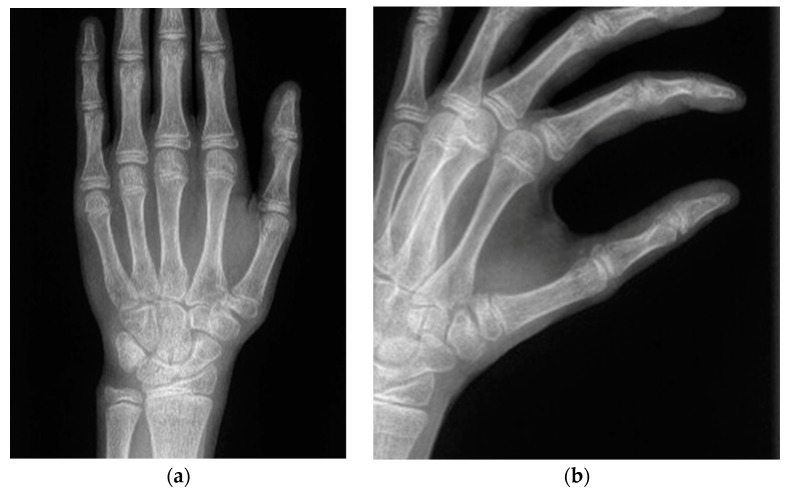
Plain radiographs of the TMC joint. (**a**) Roberts projection—strict AP view of the TMC joint. (**b**) Lateral view of the TMC joint.

- Roberts projection—strict AP view of the TMC joint. Roberts proposed forced pronation of the wrist and forearm to compensate for the obliquity of the longitudinal axis of the TMC joint in relation to the anatomical axis of the hand. This projection provides a strict profile of the interline of the trapeziometacarpal and scaphotrapezial joints and allows better visualization of the trapezium without carpal superimposition [21] (Figure 2a).
- Billings and Gedd projection—lateral view of the TMC joint [22,23]. This view allows for assessment of metacarpal displacement [24] and is very similar to this lateral view projection (Figure 2b).

In our series, this was not performed, but we suggest a first bilateral CMT radiographic evaluation to assess for CMT joint hyperlaxity or trapezius hypoplasia with symmetrical subluxation conditions.

CT and MRI scans were also performed in some patients as a follow-up method and to detect possible reluctance.

Joint hypermobility means that some or all of a person’s joints have an unusually large range of motion. This hypermobility is a very prevalent entity in children and adolescents, especially females. Realizing a correct differential diagnosis between trapeziometacarpal subluxation/luxation and hyperlaxity is key.

The diagnosis of hyperlaxity is clinical, using the Beighton and Brighton criteria. The Beighton score is measured by adding one point for each of the following:

- Placement of hands flat on the floor with legs straight;
- Left knee bent backwards; right knee bent backwards; left elbow bent backwards; left knee bent backwards;
- Left elbow bent backwards; right elbow bent backwards; right elbow bent backwards;
- Right thumb touching the forearm; left thumb touching the forearm;
- Left little finger bent backwards beyond 90 degrees; right little finger bent backwards beyond 90 degrees.

If a patient meets two major, one major and two minor, or four minor criteria with a positive clinical examination, excluding other diagnoses, he/she will be diagnosed with joint hypermobility. More detailed information on major and minor criteria can be found in Simpson’s work [25].

The presence of four or more of the described signs is a major criterion for the diagnosis of hyperlaxity. The authors ruled out the presence of hyperlaxity by evaluating the described exercises in the upper extremity.

Radiologically, Gilula’s arches [26,27,28] provide information on the assessment of normal carpal alignment on anteroposterior radiographs of the wrist. It should be remembered that radiographic carpal arches are usually altered with radial and ulnar deviation and when the wrist is not in a strictly neutral position. Hyperlaxity is difficult to assess through these Gilula lines. In contrast, bilateral stress TMC radiographs can help us to discern traumatic subluxation or dislocation from joint hypermobility. In our series, these were not necessary, as physical examination with the described criteria was sufficient.

Functionality was assessed using the quickDASH scale [29]. This was carried out at the end of each patient’s follow-up.

## 3. Results

### 3.1. Summary of the Results

Four patients were recruited between 2015 and 2023. These patients were treated at a national pediatric referral hospital. The mean age was 11.5 years (range: 9 to 13 years). Mean follow-up was 5 years (range 82–22 months). The ratio of girls to boys was 1:1. The results of the four patients mentioned above are summarized in Table 1 and described below.

Three patients underwent primary reduction in the acute phase with local anesthesia and Kalinox™ (nitrous oxide + oxygen), assisted by a nurse in the same emergency department, except for one patient who was referred from another center and whose ligament injury was later repaired by delayed surgery. Further imaging studies were required at follow-up, such as computed tomography (CT) to check for reduction or magnetic resonance imaging (MRI) to check the condition of the ligaments. The total follow-up time was 6 years after the injury.

**Figure 3:**
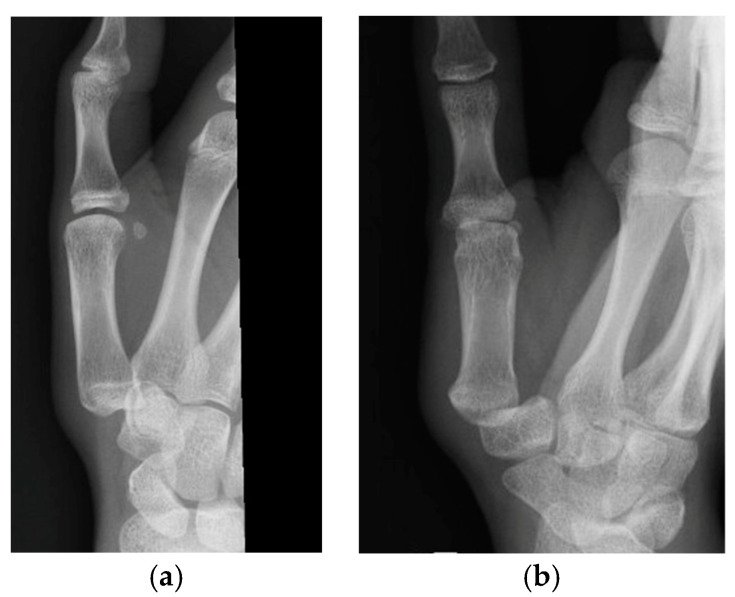
(**a**,**b**) acute TMC joint dislocation.

**Figure 4:**
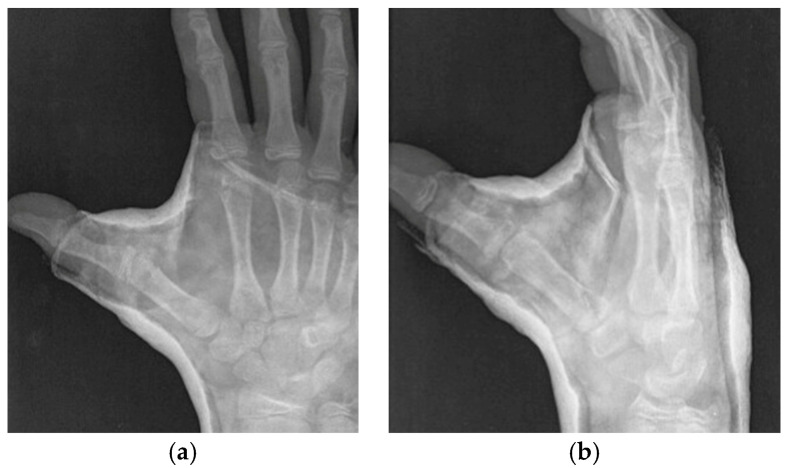
X-ray views of TMC joint after closed reduction. (**a**) AP view. (**b**) Lateral view.

**Figure 5:**
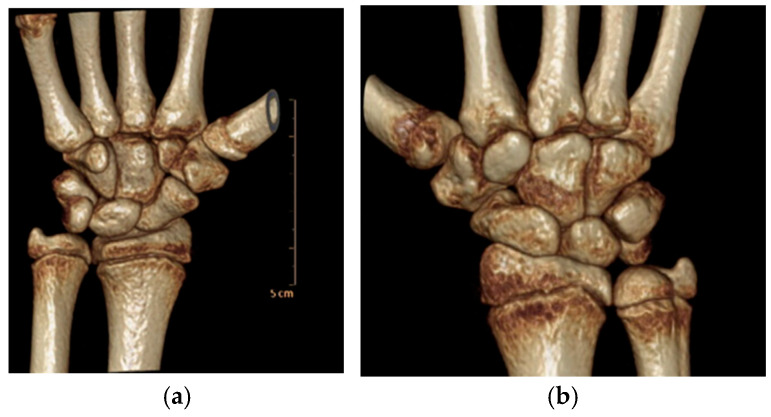
(**a**,**b**): CT was performed at two weeks (Figure 4), showing good joint congruency and no associated fractures.

The first patient was a 12-year-old female who had fallen while running with direct trauma to the thumb (Figure 3). After reduction, a short arm cast including the thumb was applied for immobilization (Figure 4). CT was performed at two weeks (Figure 5) and showed good joint congruency and no associated fractures. The cast was removed at 6 weeks with no clinical instability. Quick DASH 52.

**Figure 6:**
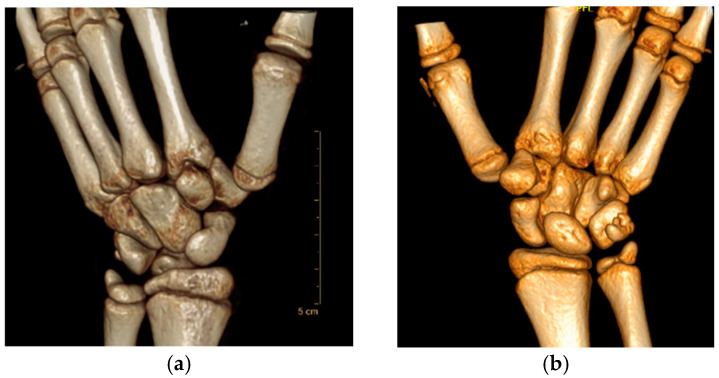
(**a**,**b**): Relapse with articular subluxation two weeks after closed reduction.

The second patient was a 13-year-old male who was immobilized in a Zancolli cast following an accidental fall. Three days after the injury, a CT scan was performed to check the reduction and showed an articular subluxation (Figure 6), which required a new manipulation and a new adapted cast. The second immediate x-ray showed a good reduction, which was confirmed by a CT scan at 2 weeks. The cast was removed at 6 weeks. Quick DASH 0.

The third case was a 9-year-old female with direct trauma to the thumb from a punch. Initial immobilization was a short arm cast including the thumb, which had to be changed after three weeks due to poor tolerance, and a protective splint was applied. CT at this time showed good reduction. A new episode after 6 months due to low-energy trauma led us to suspect instability. MRI confirmed subluxation/dislocation, and surgery was performed using the gold-standard Eaton–Litter ligament reconstruction technique with a dorsal ulnar collateral ligament anchor. In a third episode, one year after surgery, the patient presented with atraumatic TMC pain and loss of function in the contralateral thumb, and a clinical sign of hyperlaxity was now suspected. Surgery was performed with the ligament stabilization described above. Quick DASH 22.

**Figure 7:**
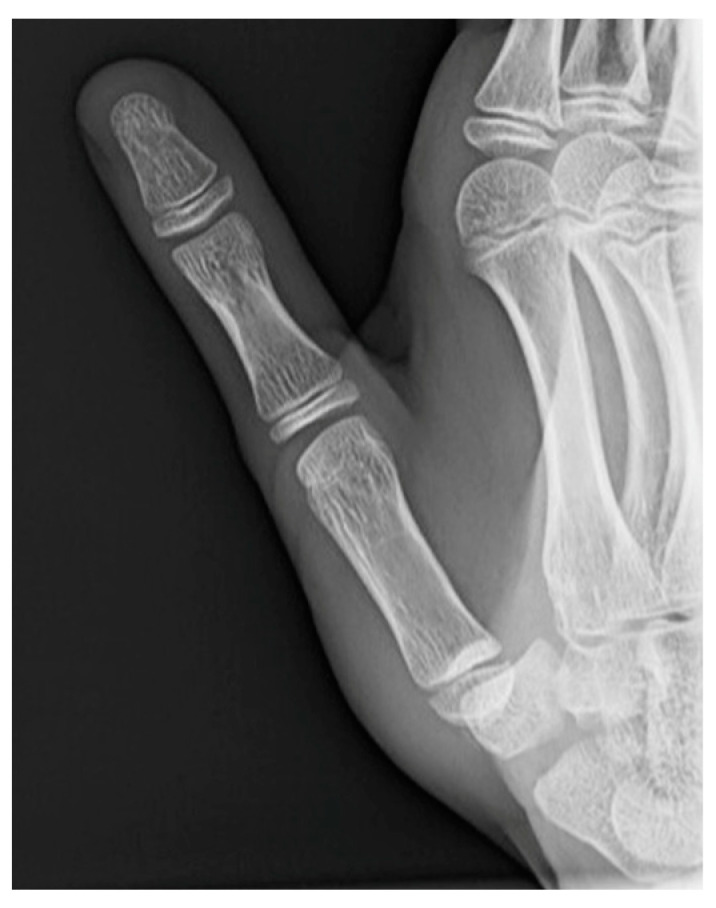
Patient referred from another center in the subacute phase.

**Figure 8:**
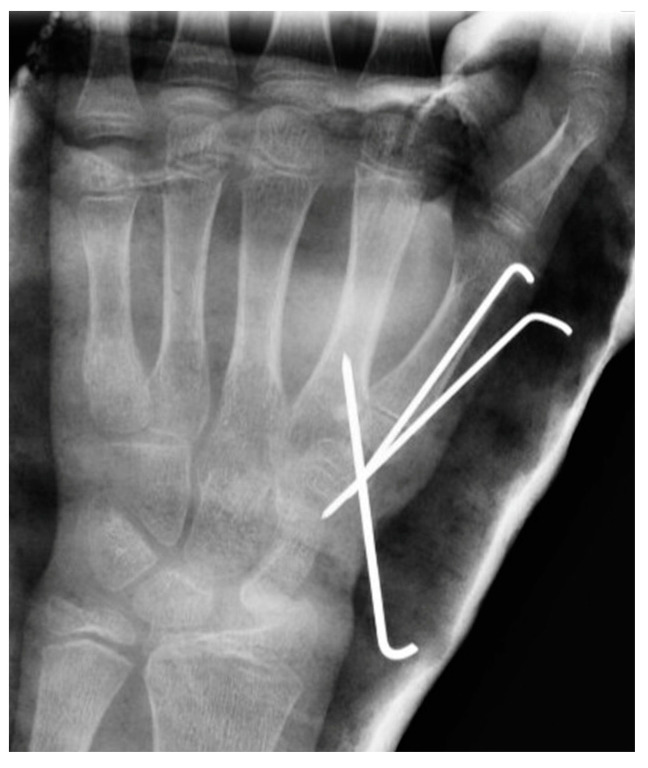
Closed reduction and percutaneous K-wire fixation.

The fourth patient (Figure 7), a 12-year-old boy, was referred from another center in the subacute phase and underwent closed reduction and fixation with percutaneous K-wires (Figure 8). Clinical, radiological (Figure 2a,b), and functional evolution was favorable, with no current symptoms. Quick DASH 0.

### 3.2. Systematic Literature Review

A review of the literature on TMC dislocations in children and adolescents [30,31,32] reveals only seven papers [2,3,15,33,34,35,36]. All of these are case reports, with the exception of Watt’s work, with two patients (Table 2).

It should also be remembered that one of the weaknesses of the systematic review is that the number of articles examined is the sample size. In our review, the total number of articles (i.e., sample size) is seven.

Therefore, for both reasons, we can state that there is no scientific evidence on the best treatment for TMC dislocations in the pediatric and adolescent age group.

## 4. Discussion

As mentioned at the beginning of this article, TMC dislocations in the pediatric age group are exceptional and may be related to hyperlaxity. For this reason, and especially in the case of dislocations associated with very low-energy trauma, self-inflicted dislocations, or recurrent dislocations, it is necessary to rule out generalized joint laxity, but also trapezius dysplasia. Whether or not hyperlaxity/trapezius dysplasia is present, a TMC dislocation must be considered an injury and treated accordingly, but, given that recurrent dislocations are not uncommon in these patients, both conditions should be identified in order to plan the most appropriate treatment and to better inform families of the prognosis.

The literature shows only seven works. This systematic review, moreover, only shows case reports and one article on two patients, so there is no scientific evidence on the best treatment for TMC dislocations in the pediatric and adolescent age group.

This paper presents the largest published series on TMC dislocation in children and adolescents.

Regarding recurrence, in our limited series, we observed reluxation in the two cases treated conservatively. Gaillard [2] reported the same complication on two occasions, the first with conservative treatment and the second with closed reduction and single-needle synthesis. As for the other articles, three other authors mentioned the failure of conservative treatment [3,33,36]. One of these patients was treated within a week of the injury; another within 8 weeks of the injury [3,33]; and for the third patient, the time between injury and closed reduction was not specified. Based on the above literature and the results of our short series, we can hypothesize a possible relationship between closed reduction and an increased likelihood of reluxation.

According to Gaillard [2], these reluxations may also be associated with immobilization periods of less than 6 weeks. This author recommends ligamentoplasty to treat late recurrences.

On the other hand, in our series, treatment with closed reduction and three-needle synthesis produced excellent results in terms of joint stabilization, mobility, and function (quickDASH). Previous authors have also reported very similar results with this closed surgical treatment [33,35].

In addition to the type of treatment, the time elapsed between injury and treatment appears to be a factor influencing the final outcome in adult patients [33]. There are no studies evaluating this variable in the pediatric age group, so this is an important area of analysis. Only Knoedler [36] recommends tenodesis surgery in cases of delayed treatment and/or recurrent dislocation for TMC instability in a pediatric patient with recurrent dislocations.

Finally, as suggested by Mc Laughin [15], we consider it important to evaluate the value of bilateral stress radiographs during the follow-up of these patients in order to dynamically assess the real stability of the joint after treatment.

## 5. Conclusions

The conclusions we can draw from this work are as follows:

- Without being able to draw conclusive data, the literature and our series report a higher rate of reluxation associated with closed reduction and plaster cast. On the other hand, there are also good results in our series and in the literature associated with this technique.
- Without being able to draw conclusive data, the literature and our series provide good clinical, radiological, and functional results that are very favorable in patients treated by closed reduction and needle fixation. Authors suggest reduction and pinning for all pediatric/adolescent cases at any time point for the acute and subacute stage.
- Without being able to draw conclusive data, in our series, open reduction and ligamentous reconstruction provide favorable results, but with significantly inferior functional results. Therefore, given this and the risk of physeal injury, we might question the suitability of this technique as a first choice, even in cases of hyperlaxity, in view of the good results of closed techniques.
- It is necessary to rule out hyperlaxity and associated trapezius dysplasia, especially in cases of very low-energy trauma and self-induced or recurrent dislocations. Whether or not there is hyperlaxity/trapezius dysplasia, TMC dislocation must be considered an injury and thus treated. However, it should not be forgotten that the coexistence of associated hyperlaxity or associated trapezius dysplasia could condition the treatment and/or evolution.
- If conservative treatment is chosen, close follow-up radiographs or, better still, CT or MRI is necessary because of the possibility of loss of reduction in initial revisions or misdiagnosis of reduction failure. Also, the literature recommends maintaining the cast for 6 weeks.

The present article can be considered a hypothesis-generating tool rather than high-quality evidence. More demanding research, such as a prospective cohort study and a randomized controlled trial, is needed to provide scientific evidence on the best treatment for TMC dislocations in pediatrics and adolescence.

## Figures and Tables

**Table 1:** Data from the four patients recruited.

Patient	Age (Years)	Sex (Female, Male)	Treatment	Loss of Reduction	CT/MRN	Thumb Intestability	Quick Dash
1	12	F	Closed Reduction + Cast Short arm cast including the thumb 6 weeks	NO	CT at two weeks shows congruency	NO	52
2	13	M	Closed Reduction + Cast Short arm cast including the thumb 6 weeks	YES *	CT at two weeks shows congruency		0
3	10	F	Closed Reduction + Cast 1. Short arm cast is intolerated after 3 weeks and changed for a brace. 2. Eaton-Litter procedure	YES	1. CT at two weeks shows congruency 2. MRN without findings	YES **	22
4 ***	12	M	Closed Reduction + KW Closed reduction and fixation with percutaneous K-wires	NO	X-ray shows favorable evolution (follow up at origin center)	NO	0

*: required remanipulation; **: required Eaton–Litter reconstructive technique for stabilization; ***: referred from another center in the subacute phase.

**Table 2:** Summary of articles published to date on TMC dislocations in pediatrics and adolescence.

Autor	N° Cases	Age (Years)	Sex	Presentation	Treatment	Clinical Results	Follow Up
Watt, 1987 [33]	2	14 12	M M	Same day 7 days	Closed reduction + KW + Cast Closed reduction + Cast	Asymptomatic and stable Painful and unstable *	- -
McLaughlin, 1998 [15]	1	11	M	Same day	Closed reduction + Cast for 6 weeks	Asymptomatic and stable	14 months
Varitimidis, 1999 [3]	1	11	M	2 months	Ligament reconstruction + KW + Cast for 6 weeks	Asymptomatic and stable	18 months
Nusem, 2001 [34]	1				Closed reduction + Cast	Asymptomatic and stable	
Gaillard, 2016 [2]	1	8	F	8 months	Ligament reconstruction + KW, after traumatic reluxation previously treated by closed methods	Asymptomatic and stable	12 months
Soldado, 2016 [35]	1	10	F	3 days	Closed reduction + KW + Cast for 6 weeks	Asymptomatic and stable	12 months
Knoedler, 2021 [36]	1	14	M	1 year	Ligament reconstruction + Mini Tightrope, (bilateral TMC) after failed closed methods	Asymptomatic and stable	5 years

*: required Eaton–Litter reconstructive technique for stabilization.

## Data Availability

Data are unavailable due to privacy.
